# Wavelet cross-correlation and phase analysis of a free cantilever subjected to band excitation

**DOI:** 10.3762/bjnano.3.33

**Published:** 2012-03-29

**Authors:** Francesco Banfi, Gabriele Ferrini

**Affiliations:** 1 Interdisciplinary Laboratories for Advanced Materials Physics (i-LAMP) and Dipartimento di Matematica e Fisica, Università Cattolica del Sacro Cuore, I-25121 Brescia

**Keywords:** AFM, band excitation, force, wavelet transforms

## Abstract

This work introduces the concept of time–frequency map of the phase difference between the cantilever response signal and the driving signal, calculated with a wavelet cross-correlation technique. The wavelet cross-correlation quantifies the common power and the relative phase between the response of the cantilever and the exciting driver, yielding “instantaneous” information on the driver-response phase delay as a function of frequency. These concepts are introduced through the calculation of the response of a free cantilever subjected to continuous and impulsive excitation over a frequency band.

## Introduction

Atomic force microscopy (AFM) has made important progresses towards the characterization of material properties at the nanoscale (elastic constants, force interactions, friction, molecular interactions, to name only a few) by means of dynamic techniques that extended the microscope capabilities well beyond simple topographic measurements [1–2]. Among the techniques developed in dynamic AFM, multimode excitation and the so called band-excitation methods have been put forward recently [3–5]. All of these techniques are based on the frequency, amplitude and phase response around one or more cantilever oscillation modes when the tip interacts with the sample surface. The temporal evolution of the amplitude, phase or frequency response is in many cases a fundamental parameter. The implementation of these techniques is based on the continuous excitation of multiple flexural cantilever modes [3–4], impulsive cantilever excitation [5] or thermal-noise excitation [6–9].

Thermal noise analysis has been performed, with the aid of wavelet transforms, to characterize the time–frequency response of a thermally excited cantilever in dynamic force spectroscopy [10–12]. In these previous works, the focus was on the time evolution of the brownian power spectral density of the tip when it is in contact with the force field of the sample surface (e.g., van der Waals, adhesion, Hertz interaction regime). However, wavelet analysis, in analogy with the classical Fourier transform, also provides *phase* information when complex functions are used as a wavelet basis.

The scope of this work is to introduce the idea that a time–frequency map of the phase difference between the cantilever response signal and the driving signal can be extracted with a wavelet cross-correlation (WCC) technique, based on the inherent phase information residing within the complex Gabor transform. This analysis has been exploited principally in the field of meteorology, oceanography and geophysical studies [13–15]. Since, to the best of our knowledge, there are no examples of WCC used in AFM, we will illustrate some examples based on the response of a damped harmonic oscillator, which in many situations is a good model for an oscillating cantilever, to different kinds of driving forces. Through the wavelet cross-correlation it is possible to quantify the power correlation and the relative phase between the cantilever response and the driving signal under reasonable assumptions [15]. In the last few years, the investigation of phase-analysis techniques [16–17] contributed to the understanding of energy-dissipation processes and elastic response in heterogeneous samples, an important topic in biological research, where the liquid environment is principally of interest. In liquids the typical cantilever Q-factor ranges from 5 [18] up to 40, for this reason we will focus our attention on the simulation of low-Q oscillators.

## Wavelet cross-correlation

The wavelet transform has shown great potential in various scientific disciplines, but it is not widespread in the context of noncontact AFM. This may be due to the absence of discussions of the practical and technical aspects of wavelet analysis relating to noncontact AFM. This article shows the use of wavelet cross-correlation by means of two simple but paradigmatic examples: The continuous and the impulsive band excitation of a free cantilever.

Before introducing the cross-correlation concept, we give a brief introduction to wavelet transform theory [19]. Wavelet analysis is based on the projection (convolution) of a discrete time series *f*(*t*) (the signal), where *t* is the time index, onto a set of continuous functions Ψ*_s,d_*(*t*) derived from the translations and dilations of a *mother wavelet* Ψ(*t*), where

[1]
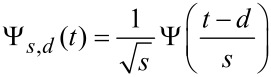


*s* and *d* are real parameters and *s* > 0. Any set of functions constructed as in Equation 1 and meeting the fundamental requirements of zero average, implying that Ψ(*t*) is an oscillating function, and rapid decay at infinity (technically Ψ(*t*) must be continuous and have a compact support; this is called the admissibility condition), are called *wavelets*.

The convolution of *f*(*t*) with Ψ*_s,d_*(*t*), at the scale *s* and delay *d*, is the *wavelet transform* (WT) of the signal *W^f^*(*s*,*d*):

[2]
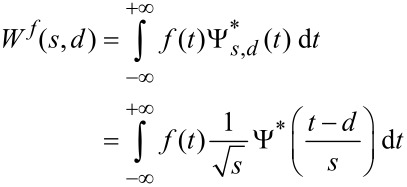


This is a continuous wavelet transform, because the parameters *s* and *d* vary continuously. The translation parameter *d* corresponds to time and the dilation parameter *s* corresponds to temporal period (or its inverse, frequency). Equation 2 expands the time series *f*(*t*) into a bidimensional parameter space (*s*,*d*) and gives a local measure of the relative resemblance of the wavelet to the signal.

The complex mother wavelet (also called Gabor wavelet or Gaussian wavelet) used in this work, as described in [10], is represented as


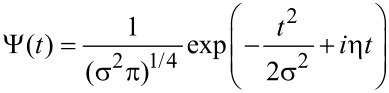


where σ controls the amplitude of the Gaussian envelope, and thus its time–frequency resolution, and η is the carrier frequency. Since the intrinsic time–frequency resolution in WT is determined by the wavelet set over which the signal is expanded, we chose a Gaussian wavelet basis because it is particularly adapted to follow signals in time, having the least spread in both the frequency and time domain and thus the best time–frequency resolution. The temporal parameter *t* in the expression of the Gabor wavelet can be regarded as a (dimensionless) discrete index and likewise σ and η are dimensionless wavelet parameters defining the wavelet shape over the discrete sampling string. The Gabor wavelet (dimensionless) center frequency at scale *s* is given by *f* = η/(2π*s*). It is possible to associate a pseudofrequency *F* (in Hz) at a scale *s* by considering that *f* is sampled with a time interval *T*, such that *F* = *f*/*T*. Therefore, the wavelet dilations set by the scale parameter *s* are inversely proportional to the frequency *F*. In the following analysis, the dimensionless wavelet parameters are chosen as σ = 1 and η = 6. This choice of parameter gives an adequate balance between time and frequency localization, which in wavelet analysis are subjected to a classical Heisenberg-like principle of indetermination (for details see [10]).

Given two time series *f*(*t*) and *g*(*t*), with wavelet transforms *W^f^*(*s*,*d*) and *W^g^*(*s*,*d*), the cross-wavelet spectrum is defined as:

[3]
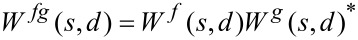


where * denotes the complex conjugate. Since the cross-correlation coefficients are complex numbers, they can be represented as *W^f^*(*s*,*d*) = |*W^f^*(*s*,*d*)|exp(Φ*^f^*(*s*,*d*)). |*W^f^*(*s*,*d*)| represents the wavelet amplitude, Φ*^f^*(*s*,*d*) is the absolute phase. Both amplitude and phase are relative to the “point” (*s*,*d*) in the frequency–time plane. The cross-wavelet power, |*W^fg^*(*s*,*d*)|, shows regions in time–frequency space where the time series have a high common power. The relative phase difference between the two time series (Φ*^f^*(*s*,*d*) = phase of *f*; Φ*^g^*(*s*,*d*) = phase of *g*), can be calculated as:

[4]
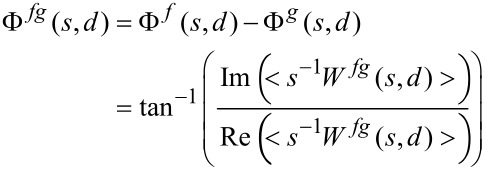


where < > represents a smoothing operator. It must be noted that this definition depends essentially on the action of the smoothing operator on the various wavelet spectra. The same situation in found in the definition of optical coherence, see [20]. For a discussion of this fundamental but rather technical aspect, see [15,21]. In general terms, a high correlation between two time series does not necessarily imply that there is any kind of connection or cause-and-effect relationship. This means that the time series can have high common power at a given time and frequency and still being uncorrelated, a problem which arises also when analyzing the correlation of signals with standard Fourier transform techniques. As an example, a correlation peak will be always present in the cross correlation between white noise and a sinusoidal signal, without implying any causal connection between the two time series. For this reason it is important to observe the phase relationship: A strong causal connection implies that the oscillations of the two series must be phase locked.

## Results

As an example to highlight the characteristics of wavelet cross-correlation, consider the case of a damped cantilever with a displacement *z*(*t*) that obeys the classical mass–spring equation

[5]
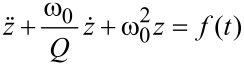


where *f*(*t*) is the driving force per unit mass.

**Figure 1:**
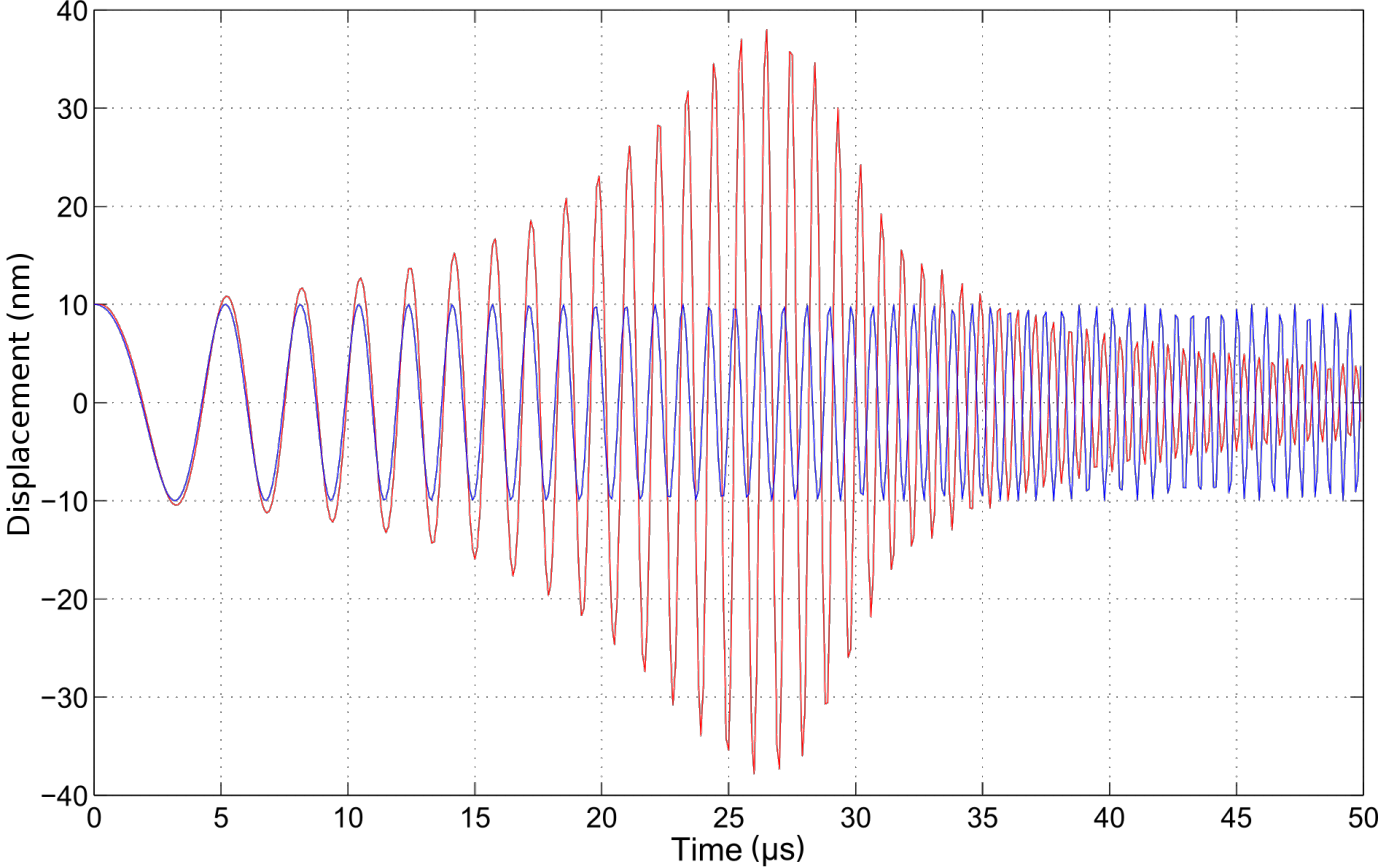
The response of a damped harmonic oscillator (red line) to a chirped driver (blue line) whose frequency is linearly swept in the interval 0.1*f*_0_ < Δ*f* < 0.9*f*_0_ over 50 μs, where *f*_0_ = ω_0_/(2π) = 1 MHz is the resonant frequency of the oscillator. The quality factor is *Q* = 4 and the initial conditions are 10 nm amplitude and zero velocity.

The above equation of motion is integrated numerically with a free resonant frequency of *f*_0_ = ω_0_/(2π) = 1 MHz, a quality factor *Q* = 4 and an excitation driving frequency that linearly sweeps the frequency interval 0.1*f*_0_ < Δ*f* < 0.9*f*_0_ in 50 μs (chirped driver). The driving function is *f*(*t*) = *z_d_*cos(ν*_d_*(*t*)*t*), where *z_d_* is the driving amplitude and ν*_d_*(*t*) the driving frequency that is linearly chirped: ν*_d_*(*t*) = *A* + *Bt*, *A* = 0.1 MHz and *B* = 0.016 MHz/μ*s*. Note that the actual instantaneous driver frequency as a function of time is the time derivative of the total driver phase, i.e., *A* + 2*Bt*. As a consequence, the resonance at *f*_0_ is excited when the instantaneous driving frequency sweeps through *f*_0_, which does *not* coincide with the frequency ν*_d_*(*t*). In Figure 1 the result of the numerical integration is shown and is compared with the driving frequency, which sweeps through the frequency band at a constant rate.

**Figure 2:**
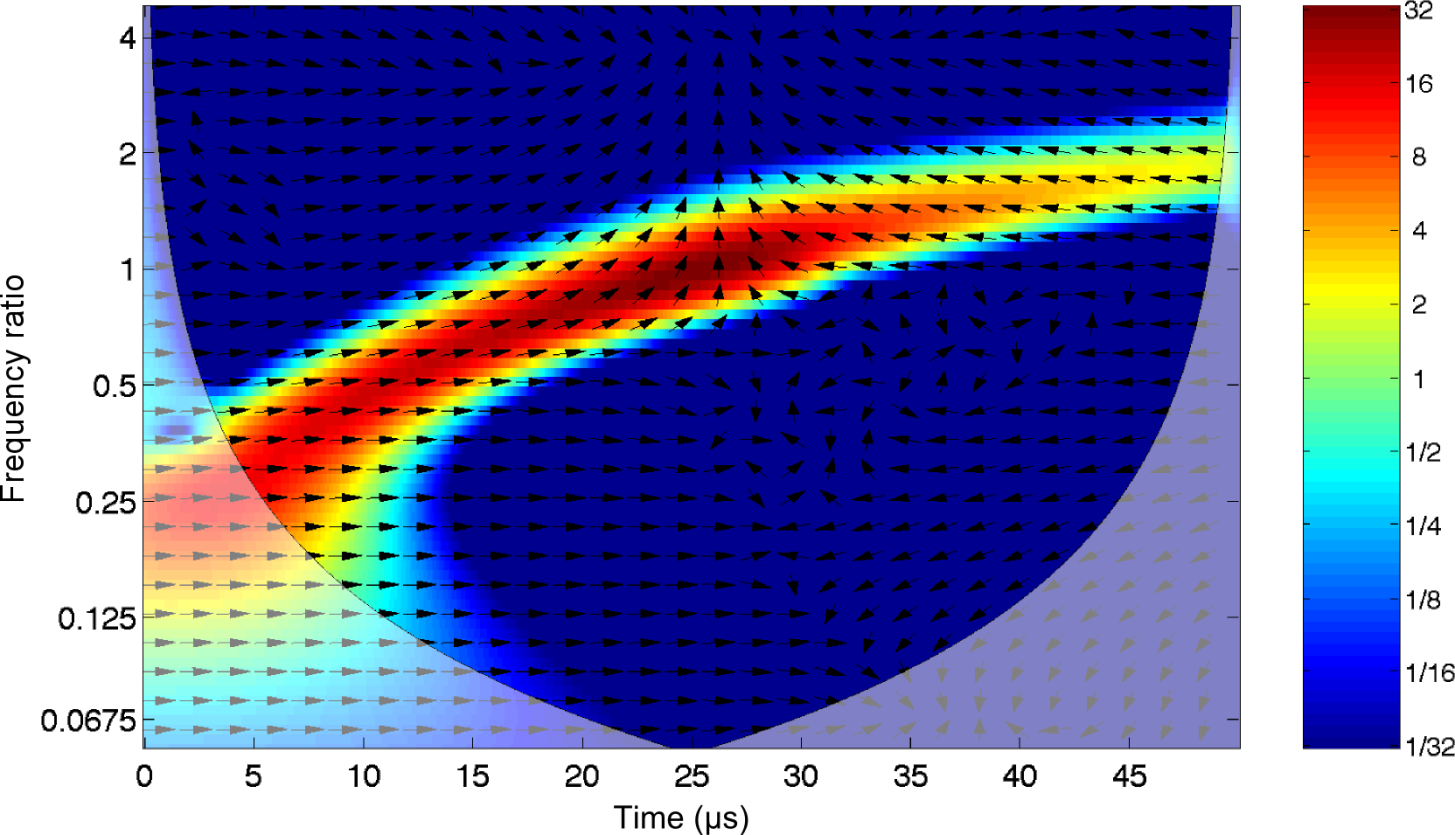
Wavelet cross-correlation between the chirped driver and the response of the damped harmonic oscillator (quality factor *Q* = 4), as shown in Figure 1. The wavelet cross-correlation coefficients (reported in the color scale) evidence the common power between the two time series in the time–frequency plane. Note that the frequency axis is represented in octaves as the base-2 logarithm of the ratio of the oscillator frequency to the resonant frequency. The color scale is proportional to the wavelet cross-correlation power and is represented in octaves. The arrows superimposed on the representation given by the color scale show the local phase difference between the oscillator and the driver. Arrow pointing right: in-phase; left: anti-phase; up: oscillator lagging behind driver by 90°. The area where edge artifacts may distort the picture are delimited by a lighter shade.

The wavelet cross-correlation analysis in Figure 2 evidences the oscillator phase relationship to the driving frequency (arrows), simultaneously with the cross-wavelet power spectral density (represented in the color scale, identifying the time-series common power), as a function of time and the instantaneous frequency [22]. The figure shows the magnitude of the wavelet cross-correlation between the two signals, *W^zf^*(ω,*t*) = *W^z^*(ω,*t*)*W^f^*(ω,*t*)^*^ and the relative phase (arrows). Phase arrows indicate the phase relationship of the oscillator to the driving sinusoid (pointing right: in-phase; left: anti-phase; up: oscillator lagging behind driver by 90°). The edge effects are delimited by continuous lines. We note that the representation in terms of the cross-correlation between the damped harmonic oscillator and the chirped driver allows us to capture more intuitively the evolution of the spectral content of the cantilever oscillations, in a way that is not possible with a traditional Fourier transform. However, the utility of this technique is even more relevant when we deal with impulsive excitation.

An excitation signal that can be used in AFM band excitation is the sinc function. It is defined as

[6]
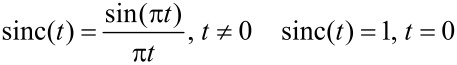


**Figure 3:**
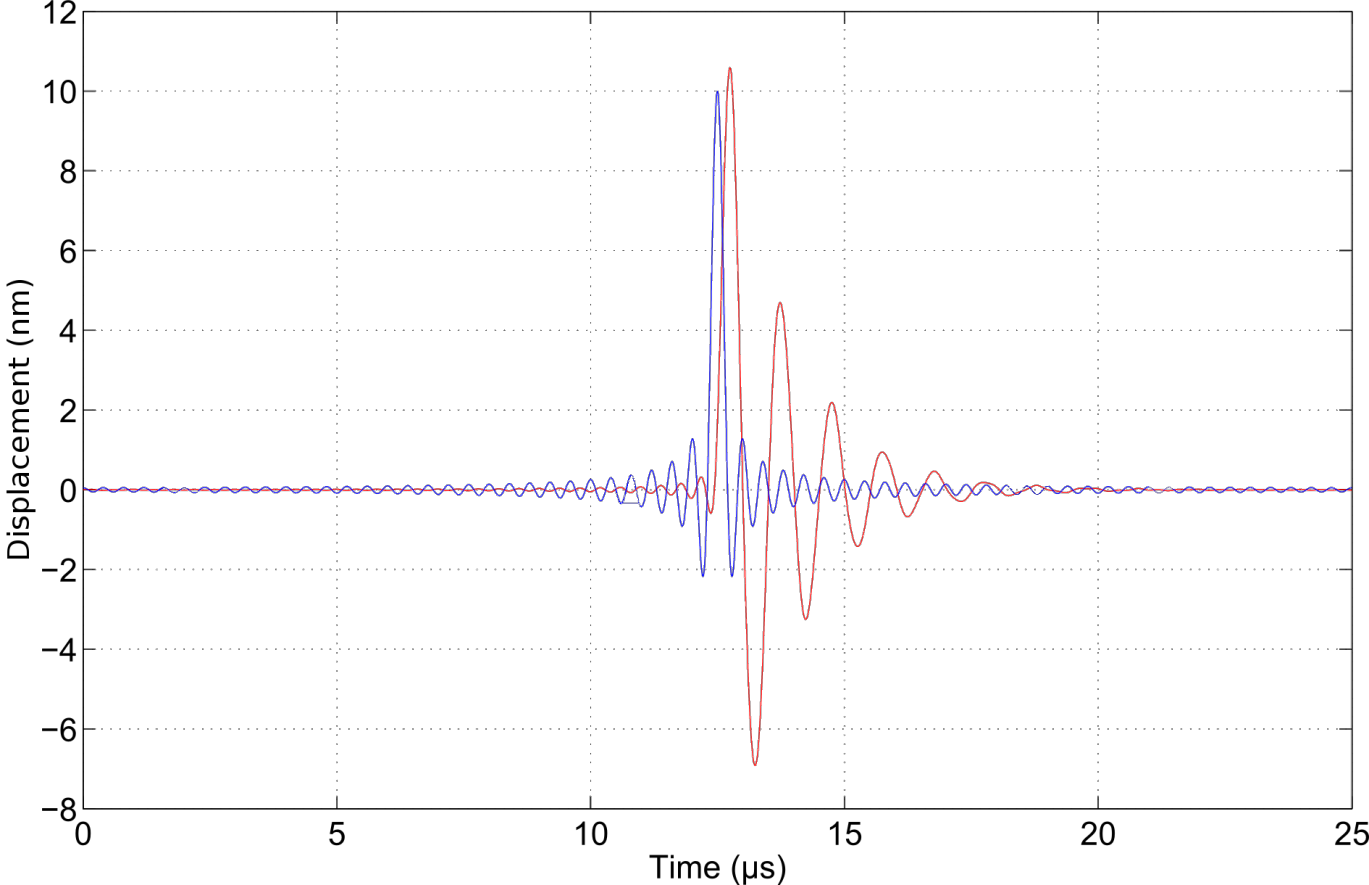
The response of a damped harmonic oscillator (red line, quality factor *Q* = 4) to a sinc driver (blue line) with an amplitude of 10 nm and a flat excitation bandwidth up to 2.5 MHz. Initial conditions are zero amplitude and zero velocity.

**Figure 4:**
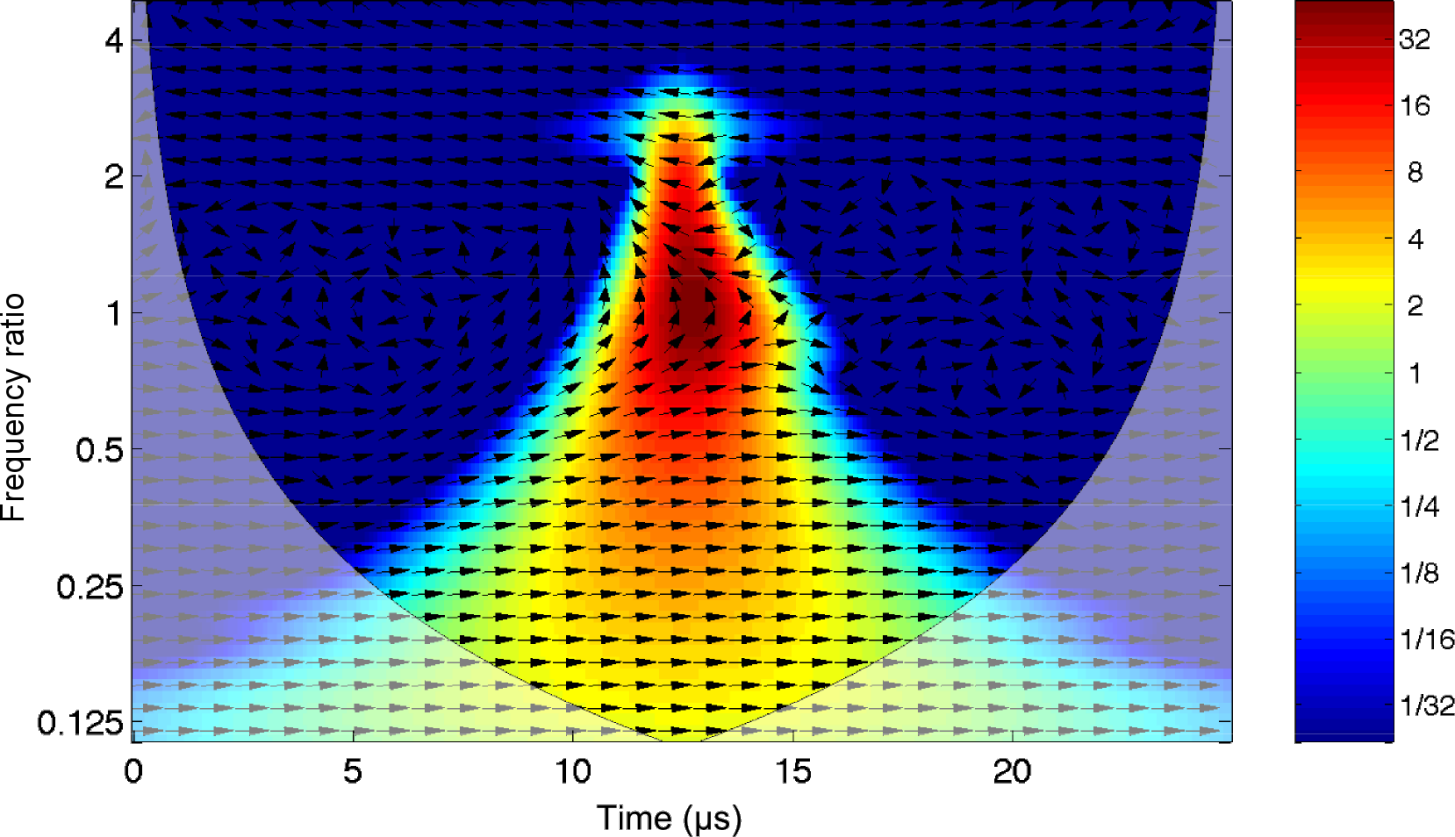
Wavelet cross-correlation between the sinc driver and the response of the damped harmonic oscillator (quality factor *Q* = 4), as shown in Figure 3.

This function is the continuous inverse Fourier transform of the rectangular pulse of width 2π and height 1. It is used as a simultaneous excitation over a limited frequency range. The time response of the damped cantilever to a properly scaled sinc function is shown in Figure 3. The response of the oscillator starts abruptly from nearly zero deflection with a finite velocity: A dynamic that is typical of impulsive forces. The wavelet cross-correlation analysis is shown in Figure 4. The spectral components have a temporal evolution peaked around the excitation pulse, as expected. To extract information from these signals, it is interesting to follow the “local” phase difference between driver and oscillator around the oscillator resonance. Below resonance the spectral components of the oscillator are in-phase with the driver, above resonance they are in anti-phase, and while at resonance they show a phase lag of π/2 with respect to the spectral components of the driver. It is important to note that the phase relations just described refer to a frequency band that has been simultaneously excited and encompasses the resonant frequency.

Although the above description of the spectral phase appears intuitive, it would not be possible to obtain it by means of a classical Fourier analysis. If the signal is not stationary, as is the case in band excitation, the squared magnitude of the Fourier coefficients measure the average energy contained in a spectral interval without tracing its effective time evolution. In this case the phase relative to each spectral component is not “local” in time, preventing its interpretation in terms of a causality relationship with a specific perturbing agent.

**Figure 5:**
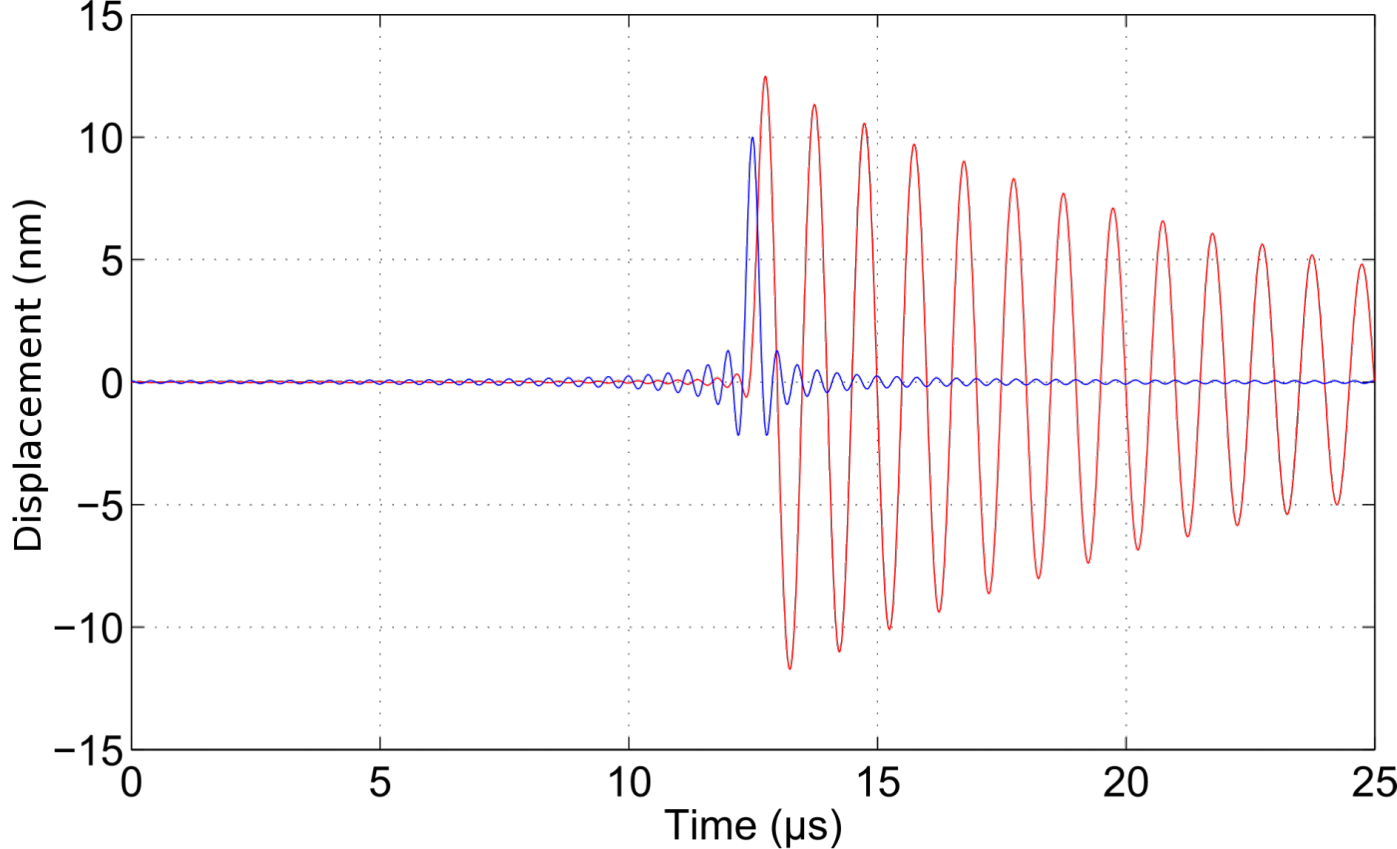
The response of a damped harmonic oscillator (red line, quality factor *Q* = 40) to a sinc driver (blue line) identical to that specified in Figure 3.

**Figure 6:**
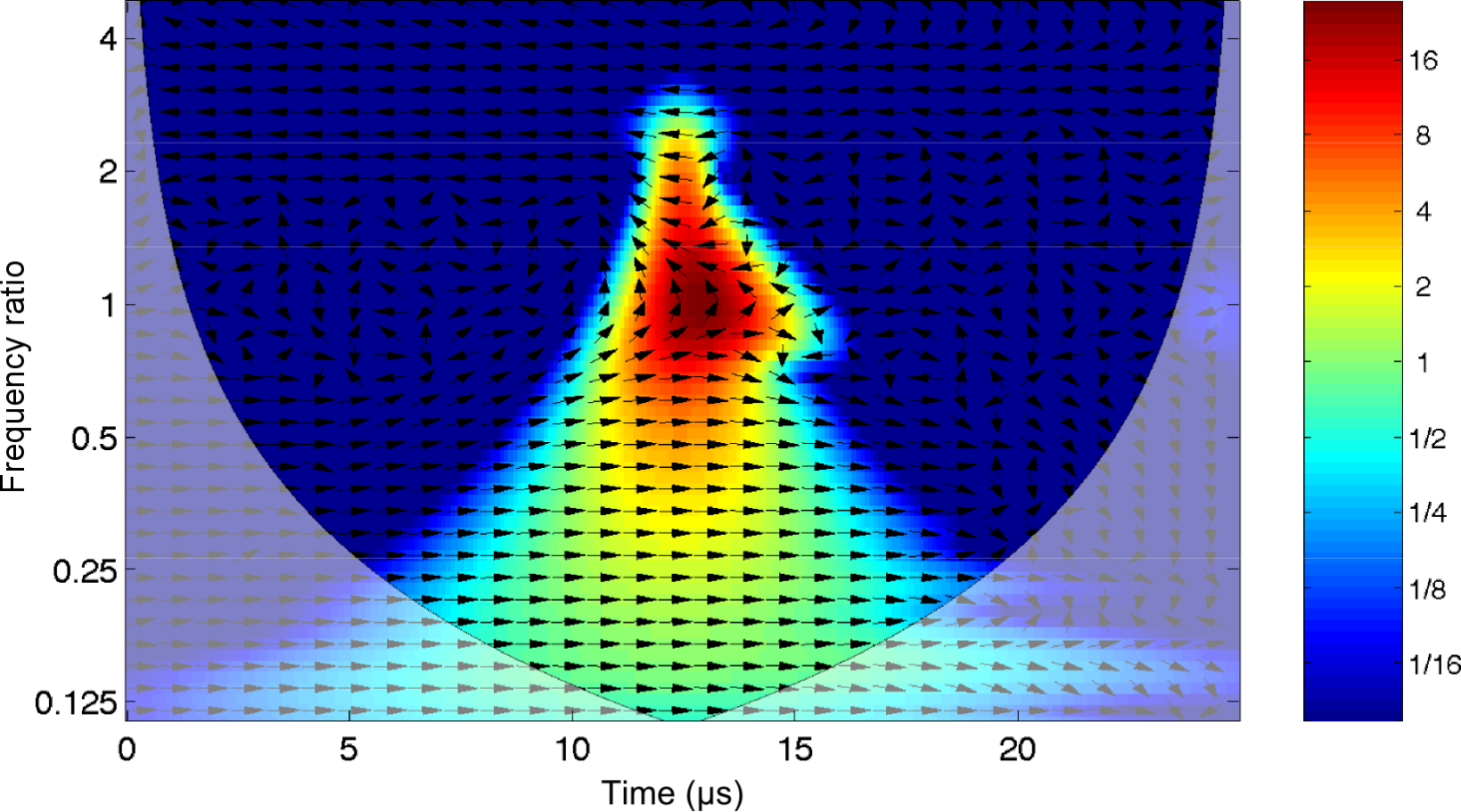
Wavelet cross-correlation between the sinc driver and the response of the damped harmonic oscillator (quality factor *Q* = 40), as shown in Figure 5.

It is interesting to note that the cross-correlation analysis allows us to separate those spectral components that are directly influenced by the driver and those relative to the subsequent evolution of the oscillator response, when the impulsive driver action has died down. We consider the same excitation as in Figure 3, but with an oscillator that has a much higher *Q*-factor, *Q* = 40. The time evolution is shown in Figure 5. We note that the initial displacement is not amplified in proportion to the Q-factor, as one would have anticipated on the basis of standard resonance amplification, as can be seen from the comparison with Figure 3. The higher Q-factor manifests as a response of the oscillator that now extends over a longer time span, well beyond the driver pulse. The wavelet cross-correlation is similar to that seen in Figure 4, because the cross-correlation is zero when the driver has decayed down and thus independent of the temporal extension of the oscillator, see Figure 6. In this case the time extent of the spectral components near resonance is increased in comparison to Figure 4 due to a less abrupt damping of the oscillaton motion.

**Figure 7:**
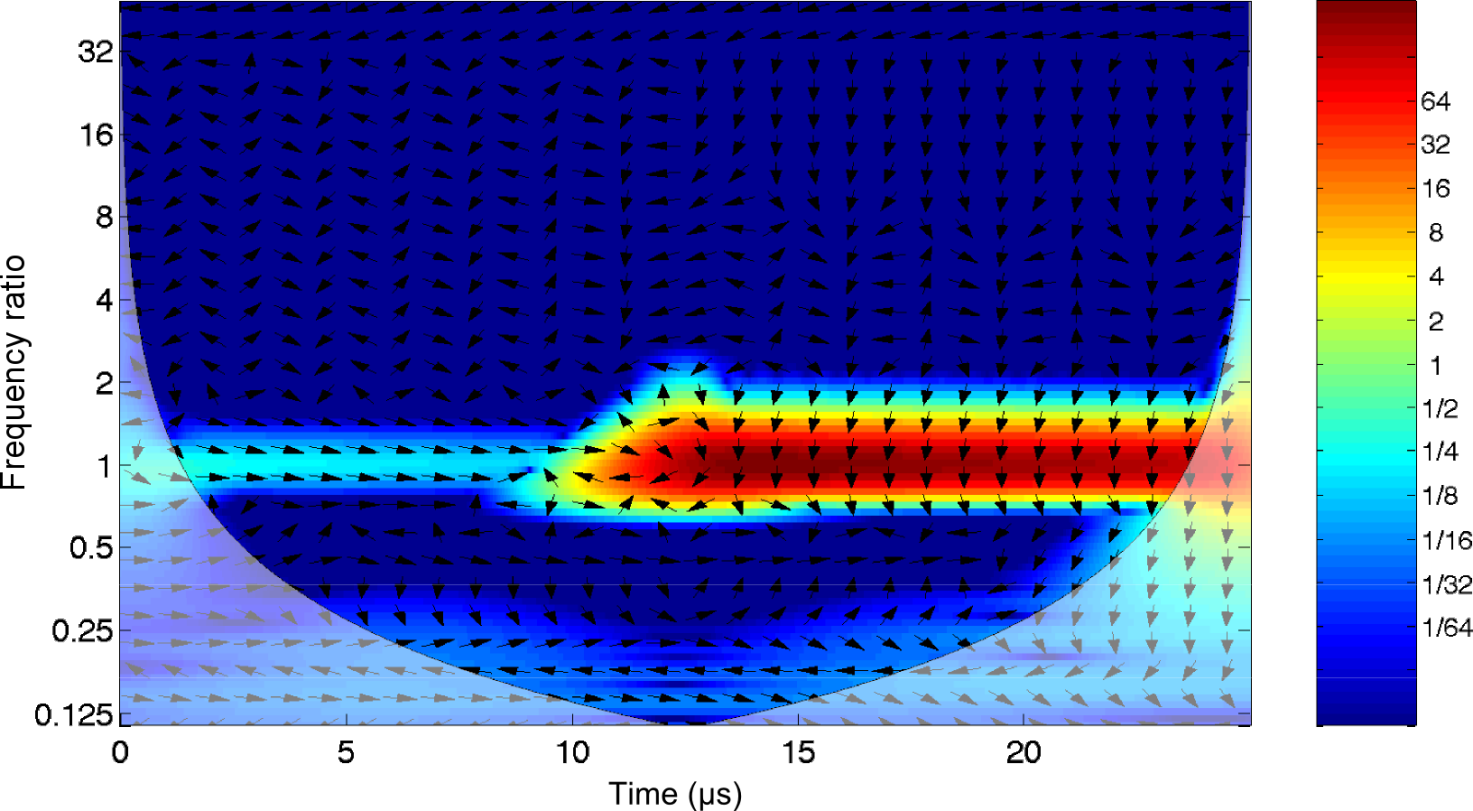
Wavelet cross-correlation between a sinusoidal reference signal at resonance and the damped harmonic oscillator response (quality factor *Q* = 40), to a sinc driver, as shown in Figure 5.

Since the oscillator signal extending beyond the driver pulse can carry useful information but is not visible in the cross correlation, an *artificial* signal can be used as a reference. The phase of the oscillator can be tracked by correlating it with a reference harmonic signal at the resonant frequency, as we demonstrate in Figure 7. In this case the oscillator phase is leading that of the reference by π/2. It is important to note here that the value of the phase difference depends on the choice of the reference signal, but its evolution in time can carry information on the interactions of the oscillator with the environment. The obvious implication that this analysis mode has on band-excitation techniques is the separation of the cantilever response into two distinct periods: An initial stage during the active driving that set the cantilever in motion and a following stage in which the undriven cantilever decays to a steady state.

### Discussion

In this section we would like to comment on how to exploit WCC and wavelet phase analysis in a practical AFM experiment, discussing the implications for the real cantilever dynamics as opposed to modeling a harmonic oscillator. A fundamental feature of wavelet phase analysis consists of measuring the phase response of the cantilever with respect to complex excitation signals (band excitations, frequency sweeps, structured pulses), and displaying the results in the time–frequency plane, with a resolution set by the Heisenberg principle, as shown in the simulations reported in Figure 2, Figure 4, and Figure 6 for a damped harmonic oscillator. This is in contrast to standard phase measurements, in which the phase response is mapped with respect to a continuous single-frequency excitation. A strategy to gain information from wavelet phase analysis relies on taking a reference “phase carpet”, corresponding to a free cantilever, for a given excitation signal. This is a time–frequency map of the phase difference between the cantilever response signal and the driving signal when the cantilever is *not* engaged in interaction. Successive excitation of the interacting cantilever provides the interaction “phase carpet”. Subtracting the interacting phase carpet from its reference, allows us to retrieve the local phase rotation, that is a function of the tip–surface interaction and the chosen excitation/driving signal. With this approach the phase rotation is measured at each frequency that resides within the excited band around a cantilever resonance and it is possible to follow its time evolution. The advantage with respect to single-frequency techniques is a more robust all-frequency characterization of the phase rotation and the possibility of connecting this information with the amplitude variation at each point in the time–frequency plane. With respect to traditional analysis, in which the spectral information is extracted with a Fourier transform, the wavelet representation disentangles the interaction spectra in the time domain. The spectral components acquire an interaction causality that is absent in the Fourier spectrum, revealing the time succession in which the phase or the amplitude at a specified frequency has been altered by the interaction. In certain cases this information may be of great utility, for example to enable correlation of phase-jumps with the interaction processes, that usually have time-scales that are a fraction of the oscillation period. It is foreseen that in similar cases the wavelet analysis could track dynamics otherwise not visible in a Fourier spectrum because of the superposition of spectral contributions generated at different times.

In amplitude-modulation AFM (tapping mode) wavelet analysis is useful to track the time evolution of the nonlinearities in tip–surface dynamics. The wavelet analysis allows one to follow more than a single flexural mode simultaneously [10–11] and the eventual harmonics due to a nonlinear response, characterizing their time evolution. Regarding the phase response, we expect that nonlinear interaction will produce phase discontinuities in the WCC between the driving signal and the cantilever response, whose temporal dynamics should be accessible. As an example, the spectral response of a cantilever in liquid excited at its first flexural resonance, and which impacts on a sample, is controlled by the elastic parameters of the sample and determines the degree of excitation of the higher flexural modes [17]. The cantilever spectral distribution upon impact, captured with wavelet amplitude and phase analysis, is thus a fingerprint of the material properties. This information can be used, at the very least, to determine compositional contrast.

A final remark is due concerning the effect of noise (thermal and environmental noise) on wavelet analysis while processing data collected under normal AFM operating conditions. One might expect noise to be a limiting factor when performing wavelet analysis, due to the fact that the wavelet analyzes the signals for a shorter time and therefore loses the averaging effect present in traditional Fourier spectra. Regarding environmental noise, it has been demonstrated that by using only thermal excitation it is possible to retrieve useful information from force spectroscopy [11] with a *single* approach curve under standard operating conditions. Regarding the thermal noise, the excitation signals must have amplitudes exceeding that of the thermal noise, because averaging is limited or absent. In this case, the choice of the excitation amplitude depends on the type of cantilever, on its quality factor and on the parameters to be measured. We anticipated that only extremely low amplitude excitations should have portions of the time–frequency map rendered useless below the noise floor. Further (ongoing) studies will be necessary to gain insights into the limitations of wavelet analysis.

## Conclusion

The application of wavelet analysis to interacting cantilevers is a promising route to the characterization of material properties on the nanoscale. The wavelet correlation technique allows one to measure the phase relationship between driver force and cantilever response in complex excitation schemes. The complete time–frequency picture of the phase evolution can be exploited as an important tool to characterize material response and tip–sample interactions. The wavelet correlation analysis sets into a different perspective the AFM techniques, which have been analyzed so far only in terms of Fourier transform.
